# The effect of stem cell therapy and comprehensive physical therapy in motor and non-motor symptoms in patients with multiple sclerosis: A comparative study

**DOI:** 10.1097/MD.0000000000021646

**Published:** 2020-08-21

**Authors:** Alia A. Alghwiri, Fatima Jamali, Mayis Aldughmi, Hanan Khalil, Alham Al-Sharman, Dana Alhattab, Ali Al-Radaideh, Abdalla Awidi

**Affiliations:** a Department of Physiotherapy, School of Rehabilitation Sciences; b Cell Therapy Center, the University of Jordan, Amman; c Department of Rehabilitation Sciences, Faculty of Applied Medical Sciences, Jordan University of Science and Technology, Irbid; d Laboratory for Nanomedicine, Division of Biological & Environmental Science & Engineering (BESE), King Abdullah University of Science and Technology (KAUST), Thuwal, 23955-6900, Saudi Arabia; e Department of Medical Imaging, Faculty of Applied Medical Sciences, Hashemite University, Zarqa, Jordan; f School of Medicine, the University of Jordan, Amman, Jordan.

**Keywords:** mesenchymal stem cells, multiple sclerosis, physical therapy, quality of life

## Abstract

**Introduction:**

People with multiple sclerosis (PwMS) experience a wide range of disabilities which negatively impact their quality of life (QOL). Several interventions have been used in PwMS such as medication, physical therapy exercises and stem cell therapy to improve their QOL. However, there is a limited evidence on the benefits of combining interventions. The purpose of this study is to explore the effect of combining physical therapy exercises (PTE) and Wharton Jelly mesenchymal stem cell (WJ-MSCs) injections on motor and non-motor symptoms versus each intervention alone in PwMS.

**Methods:**

Sixty PwMS will be allocated to either PTE, WJ-MSCs, or a combined group, followed up for 12 months and examined using a comprehensive battery of measures. Participants in the PTE group will receive 2 sessions per week of a supervised exercise program for 6 months followed by a home exercise program for another 6 months. The WJ-MSCs group will receive 3 WJ-MSCs injections in the first 6 months then they will be encouraged to follow an active life style. The third group will receive both interventions.

**Discussion:**

This study will aid in a better understanding of the combined effect of physical therapy and mesenchymal stem cell therapy. The results from this proposed study may reduce disability, improve QOL in PwMS, and consequently, reduce the cost associated with the life-time care of these individuals worldwide.

**Trial registration number:**

NCT03326505

## Abstract 2

Supplemental Digital Content is available in the text

## 1 Introduction

Multiple sclerosis (MS) is a progressive inflammatory neurodegenerative disease characterized by a variety of disabling motor and non-motor symptoms.^[1]^ Motor symptoms such as muscle weakness and spasticity often lead to gait abnormalities and balance impairments.^[2]^ Non-motor symptoms such as cognitive dysfunction, depression, fatigue, and sleep disturbances are also common in these individuals.^[3]^ These symptoms together can result in a reduction in several quality of life (QOL) indices, including physical function, psychological well-being, self-care, work ability, interpersonal relationships, and reduced ability to perform daily activities.^[1,3,4]^

MS can be found all over the globe, with an estimated 2.3 million people diagnosed annually worldwide (33 cases per 100,000 people). The prevalence of MS is the highest in North America and Europe while Sub-Saharan Africa and East Asia have the lowest prevalence.^[5]^ In Jordan, data from 2004 to 2005 indicated a prevalence of 39/100,000 in the capital, Amman and 38/100,000 in the northern city of Irbid, which is considered a medium to high risk similar to western countries.^[6]^

Participation in physical therapy exercises (PTE) should start soon after the MS diagnosis, as research has shown that PTE delivers favorable outcomes in a variety of motor and non-motor symptoms in people with MS (PwMS), even in those who are not severely disabled.^[7–10]^ Balance, mobility, spasticity, fatigue, and overall QOL have been all found to improve from participating in PTE in the MS population.^[7–10]^ Unfortunately, the lack of awareness about the role of physical therapy in the management of PwMS might be the reason why PwMS do not receive adequate physical therapy services in Jordan.

Regenerative medicine using stem cell therapy injections has also proven its efficacy in numerous preclinical and some clinical studies in halting the neurodegenerative and neuro-inflammatory aspects of MS disease.^[11–13]^ One of the attractive stem cell therapy types is umbilical cord mesenchymal stem cells using Wharton Jelly (WJ-MSCs) due to their accessibility, high proliferation rate and their unique neuroregenerative potential as compared to bone marrow or adipose tissue derived MSCs.^[14]^ The WJ-MSCs was used in several studies for different aims such as cartilage repair and bone regeneration.^[14–16]^ The Cell Therapy Center (CTC) at University of Jordan has demonstrated the safety and efficacy of this therapy for the past years in the setting of clinical trials, the first of which was for PwMS who had not responded to any available pharmaceutical agent.^[11]^

While the effect of mesenchymal stem cell therapy on PwMS has been studied before,^[11]^ and PTE shown to be effective in PwMS,^[17]^ there is a limited evidence on the combined effect of WJ-MSCs and PTE on a wide range of symptoms in PwMS. Therefore, this study will be the first to assess the effect of a combined treatment intervention protocol (WJ-MSCs and PTE) that will eventually aid in a better understanding of the combined effect of 2 different treatment interventions versus when administered alone, in relation to motor and non-motor symptoms in PwMS. The findings of the study will emphasize the important role exercises have when added to regenerative medicine on managing common symptoms in PwMS. We also hope that the results of this study help guide clinicians and primary care givers on including physical therapy services soon after the diagnosis of MS to improve the QOL of these individuals.

### 1.1 Objective

The purpose of this study is to explore the effect of PTE and WJ-MSCs injection versus PTE alone or WJ-MSCs alone on motor and non-motor symptoms in PwMS. We hypothesize that PwMS who receive the combined treatment of WJ-MSCs and PTE will demonstrate significant improvements on motor and non-motor symptoms compared to those who receive either WJ-MSCs alone or PTE alone.

### 1.2 Trial design

**Figure 1:**
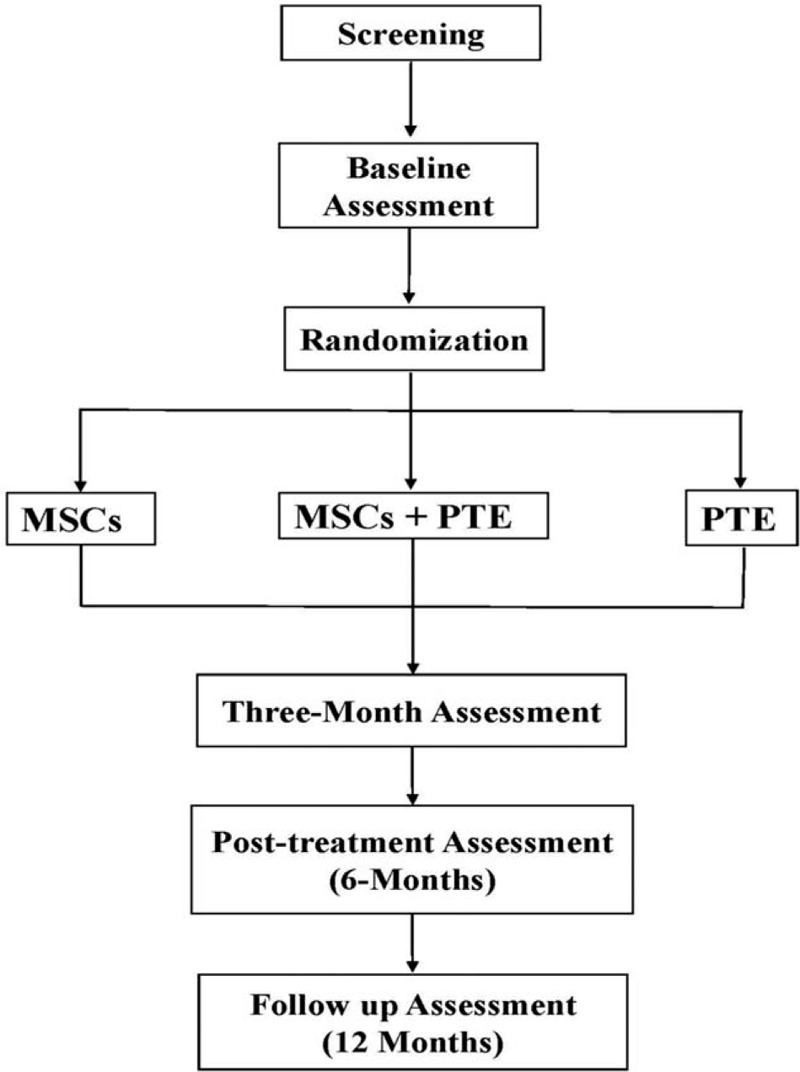
Flow chart demonstrating the study procedures from screening through study completion.

The proposed study utilizes a randomized controlled design to examine the combined effect of a 6-month program on motor and non-motor symptoms in PwMS. This study is single-blinded where the researchers involved in the assessments are blinded to the allocation of the participants to each group. We aim to recruit 60 participants with MS (20 in each group) that will be randomly allocated to 1 of 3 groups using a computer-generated software to either a combined treatment group (PTE + WJ-MSCs), PTE alone control group or WJ-MSCs alone control group after going through a battery of comprehensive assessments (Fig. 1).

## 2 Methods

### 2.1 Study setting and recruitment

Participants will be recruited from the MS registry list at the CTC facility at University of Jordan, from personal referral from consented participants, area physicians, or study personnel, and advertisement on social media.

### 2.2 Eligibility criteria

Interested participants will be checked for the inclusion/exclusion criteria where they will undergo a thorough neurological exam performed by a neurologist to generate the expanded disability status scale (EDSS), and a trained research personnel will perform the mini mental state examination.^[18]^ A screening form is also filled out for every participant that includes demographics, general history of the disease, past, and current medication intake and relevant clinical aspects of the disease. Participants will be selected based on the following eligibility criteria:

Inclusion criteria:

(1) Males and females.
(2) Eighteen years and older.
(3) Written informed consent prior participation.
(4) Independent in ambulation even with the use of an assistive device.
(5) All types of MS.
(6) EDSS score < 6.5.
(7) mini mental state examination score ≥ 24.

Exclusion criteria:

(1) Uncorrected vision loss.
(2) History of a nervous system disorder other than MS.
(3) Severe physical, neurological, or sensory impairments that would interfere significantly with testing.
(4) Developmental history of learning disability or attention-deficit/hyperactivity disorder.
(5) Relapse and/or corticosteroid use within 4 weeks of assessment.
(6) Acute ischemic cardiovascular event or coronary artery bypass surgery less than 3 months.
(7) Uncontrolled blood pressure with medication (blood pressure (BP) > 190/110 mm Hg).

### 2.3 Interventions

#### 2.3.1 Mesenchymal stem cells (control group 1)

The stem cell treatment protocol for all participants in the WJ-MSCs only group or the combined group consists of using cryopreserved allogenic WJ-MSCs donor. The WJ-MSCs group receive 3 injections. The first one contains 100x10^6^ cells intrathecally and 50 x10^6^ cells intravenously. After 1 month, the second injection is administered with the same content as the first injection. The third intrathecal injection contains 8 to 10 mL of the WJ-MSCs’ conditioned media after 3 months of the first injection. The WJ-MSCs is administered by an internal medicine specialist or the neurologist himself. Prior to the intrathecal injection, the same volume of MSCs injected is withdrawn as cerebrospinal fluid (CSF) in order to maintain the CNS pressure and decrease the post-intrathecal injection headache.

Prior to each treatment, patients’ vital signs are measured at the CTC “ICU equipped” transplantation room. The participants are kept under observation for an hour post injection for any immediate adverse events. Any related adverse event is noted by the CTC nurse, 24 hours, 1 week, and 1 month post treatment through direct communication with the participant.

##### 2.3.1.1 Isolation and expansion of WJ-MSCs

The consenting umbilical cord donor is a lady in labor at the “Al-Amal Maternity Hospital” with blood group O who has been screened for infectious agents (HIV, Syphilis, Hepatitis B and C). Isolation of MSCs from the Wharton jelly is performed using the explant method.^[19]^ All steps are conducted according to good laboratory practice and good cell culture practice principles at the CTC clinical tissue-culture lab. A cell bank generated from a single donor expanded to a clinical grade provides sufficient number of cells to be transplanted to all patients eliminating the donor variability in interpreting treatment outcomes.

The maternal umbilical cord is rinsed with (PBS) (pH7.4) and cut into 2 cm-long pieces and plated in round tissue culture dishes. The explants are allowed to attach for about 15 minutes, then culture medium is gently added to the plates and incubated for 8 days in humidified 5% CO^2^ incubators. Cells are fed with fresh medium every 3 days until individual colonies reach 70% to 80% confluence. After the primary passage, cells are detached using the xenogenic-free10xTriple E (Thermo Life Technologies, ThermoFisher, MA. WJ-MSCs are reseeded at a density of 4 × 10^3^ cells/cm^2^ in 175 cm^2^ flasks and subsequently moved to double layer Nunc Cell Factory Systems (Nunc, Thermo Scientific, Waltham, MA) to reach clinical numbers at passage 3. Cells are cryopreserved and adequate number are thawed and allowed to proliferate to be ready as treatment at passage 4. The conditioned media of WJ-MSCs are stored at –800° C Freezers at the CTC to be used for the third injection.

The expansion culture medium consists of the following: α-modified minimum essential medium (α-MEM; GIBCO, ThermoFisher, MA) supplemented with 10% platelet lysate, 1% (w/v) Penicillin/Streptomycin (P/S) and 2 mM L-glutamine (GIBCO, ThermoFisher, MA). The culture medium is changed twice a week. The quality of WJ-MSCs is examined throughout the expansion steps visually and microscopically for any fungal or bacterial contamination and using Q-PCR for any mycoplasma contaminant prior to patient administration.

##### 2.3.1.2 Characterization of WJ-MSC

Characterization of the patients’ expanded MSCs is in accordance with the International Society for Cellular Therapy (ISCT) recommendations.^[20]^ Cells are observed microscopically for the spindle shape and attachment to tissue culture flasks. Flow cytometry is performed to confirm the expression of CD73, CD90, and CD105 surface molecules at a percentage of at least 95% and the absence of CD34, CD45, CD14, and CD3 surface markers at no more than 5%. In addition, the differentiation assessment into adipogenic, osteogenic, and chondrogenic lineages is performed according to the differentiation media manufacturing recommendations; StemPro differentiation media (GIBCO, ThermoFisher, Waltham, MA).

#### 2.3.2 Physical therapy exercise) program (control group 2)

The PTE program utilized in this study is partly based on 2 previous published exercise programs that were found to be effective in improving balance and mobility in PwMS.^[7,21]^ Following the physical activity guidelines for adults with MS,^[22]^ we will further add 30 minutes of aerobic exercise to the program. Additionally, we will also follow the guidelines of having a minimum 2 sessions per week of physical activity for PwMS. The PTE program consists of a mixed program including the following types of exercises: balance, resistance, flexibility, and aerobic exercises performed 2 times per week for a total of 6 months. The intensity of the exercise will be progressed throughout the 6 months’ period and consists of 3 stages of progression for the resistance/flexibility and balance programs, and 2 stages of progression for the aerobic exercise program. Participants will be progressed to each stage depending on their tolerance and performance level of the exercises. Each session will take 60 to 80 minutes to finish, and will be supervised by a trained physical therapist (PT). Exercise will be performed in a well-equipped lab space at the CTC at University of Jordan. Following the 6 months supervised program, participants will continue the remaining 6 months of the study period performing a home exercise program (HEP).

The PTE session is divided as the following: pre-exercise vital signs measured using a digital HR/BP monitor, 5-minute warm-up exercises, 10 to 15 minutes of strength and flexibility exercises, 10 to 15 minutes of postural balance exercises, 40 minutes of aerobic exercise on a recumbent stepper machine and a 5-minute cool-down. Vital signs will be taken again post-exercise. Resting periods will be given to each participant before progressing to other forms of exercises and as necessary to decrease levels of fatigue. Participants will be allowed to choose the appropriate days/times to do the sessions upon their convenience but will be encouraged to choose the session every other day to allow a day rest in between. Vital signs, comments, and progress of each session will be documented for every participant using weekly exercise logs.

The warm up/cool down exercises that will be performed before and after the PTE will each take around 5 minutes; all exercises are to be done for 3 to 5 repetitions. The PT will demonstrate and supervises the exercises for the participants. The warm up/cool down exercises include chin tucks, arm circles, shoulder shrugs, marching in place, hamstring stretch, heel cord stretch/calf stretch, knee to chest, heel raises/toe raises, and mini-squats (a chair or wall in front can be used if needed for support). The remaining supervised exercises will be performed in the following order:

##### 2.3.2.1 Resistance and flexibility exercises

The following guidelines are followed across the 3 stages: A list of 10 different exercises is given to the participant. The participants are allowed to choose 5 exercises out of the 10 exercises each session with the help of the PT. In case the participant was not able to perform 1 of the exercises, the PT chooses another 1 from the remaining 5 and so on. The participant perform the other 5 exercises the next session and alternate every session accordingly. For the exercises that requires resistance, if the participant cannot perform any of the exercises against resistance using cuff weights, Thera band, etc. The participant is allowed to perform the exercise without resistance until he/she feels comfortable with it and can perform it successfully pain free. The PT will add resistance when participant perform the exercise successfully and pain free. If the participant cannot perform the required number of repetitions, the PT decreases the frequency to the participants’ ability and increase accordingly until reaching the desired outcome. The PT records the exercises/intensity/frequency performed by the participant on the weekly exercise log every session. The resistance and flexibility exercises through the 3 stages are listed in detail in Supplemental Digital Content (Appendix 1).

##### 2.3.2.2 Balance exercises

The following guidelines should be followed across the 3 stages: a list of 10 different exercises is given to the participant. The participants are allowed to choose 5 exercises out of the 10 exercises each session with the help of the PT. In case the participant was not able to perform 1 of the exercises, the PT chooses another 1 from the remaining 5 and so on. The participants perform the other 5 exercises the next session and alternate every session accordingly. For the exercises that requires challenges, if the participant cannot perform challenging exercises using: balance board, Swiss ball, dual tasking…etc. The PT will allow the participant to perform the exercise without the challenge until comfortable with exercise, and then add the challenge when participant perform the exercise successfully and safely. The PT will add resistance when participant perform the exercise successfully and pain free. If the participant cannot perform the required number of repetitions, the PT decreases the frequency to the participants’ ability and increase accordingly until reaching the desired outcome. The PT records the exercises/intensity/frequency performed by the participant on the weekly exercise log every session. The balance exercises through the 3 stages are listed in detail in Supplemental Digital Content (Appendix 2).

##### 2.3.2.3 Aerobic exercise

The following guidelines are followed across the 2 stages: exercise intensity in stage I is 50% to 59% of heart rate reserve for 3 months; increased to stage II 60% to 69% of heart rate reserve for remaining 3 months. Exercise session total time is 40 minutes on the recumbent stepper supervised by the PT including 5 minutes warm up, 30 minutes aerobic training, and 5 minutes’ cool down. Aerobic exercise is performed on the same recumbent stepper used for the submaximal testing during the baseline assessment (Model SCIFIT-StepOne). The handles and seat is adjusted according to the participant's arm and leg length prior to the exercise session (taken from the submaximal test performed at baseline). Pre and post exercise vital signs (HR and BP) are taken. Heart rate is monitored during the exercise using polar heart rate monitors. Five minutes’ warm-up and cool down on the recumbent stepper is set at 25 watts load at a comfortable pace. Exercise intensity during the 30 minutes’ period is increased to the prescribed target heart rate range that is initially calculated using the Karvonen formula.^[23]^ Intensity is adjusted according to physiologic response but not to exceed the target heart rate range during the 30 minutes’ period. Rate of perceived exertion is monitored before and after the aerobic training period using the Borg scale. During the 30 minutes of exercise, each participant is instructed to keep their stepping pace at 100 steps/min that would be visible for them on the machine monitor. However, each participant is allowed ± 5 steps (95–105 steps/min) during the exercise. The intensity used, vital signs, rate of perceived exertion, and any comments or changes during the session is recorded on a weekly exercise log. After the cool down, participants are allowed ample time to rest before they leave.^[24]^

##### 2.3.2.4 Home exercise program

The HEP focuses mainly on the home use of an exercise DVD that was specifically developed for people with movement disorders and available in the Arabic language. Details of the exercise DVD and its content are published elsewhere.^[25,26]^ In brief, this DVD focuses on the main components of fitness introduced by an acting lady and a gentleman in 5 main sections. The first section is focused on warm up and flexibility activities; the second, third, and fourth sections focus on strength, flexibility, balance and endurance exercises, and training on performing functional tasks of sit to stand, stepping up onto stairs, and getting on and off the floor; and the fifth section focuses on relaxation, stretching, and breathing techniques. In addition to these main 3 sections, the DVD includes a list of precautions, equipment required, and postural instructions.

During the HEP, participants are instructed to perform the exercises 2 times a week using the exercise DVD. After the end of the first 6 months of the intervention, the participants will receive 1 instructional session in the CTC from a trained PT. In this session participants are introduced to all exercises in the exercise DVD; the therapist observe the participant while performing the exercises and provide feedback whenever is needed. There is a total of 29 different exercises in the DVD, the participants are encouraged to perform all of them. However, the participant is allowed to choose which exercises to perform if any of the exercise were found difficult to execute or if it was not feasible to perform at their home. Participants should perform at least half of the exercises in the DVD. Each participant will be provided with the DVD along with a hardcopy manual of the exercises. In addition, potential benefits and risks of performing the exercises are discussed. During the instructional session, participants are also instructed on how to progress their exercises by gradually increasing the number of repetitions while decreasing the number and length of rest breaks and increasing the level of exercise progression. In addition, participants will be contacted by the PT every month via phone calls to assess their compliance to the program and document any barriers to exercise of they have any. A predetermined protocol for these instructional sessions is provided in Supplemental Digital Content (Appendix 3). For the remaining period of the program, participants are asked to perform the 2 exercise sessions per week independently at home. Participants are asked to keep an exercise diary for their HEP exercises to assess their compliance to the HEP program. Participants will return the exercise diary at their 1-year post assessment appointment. Supplemental Digital Content (Appendix 4).

#### 2.3.3 Combined treatment group (PTE and WJ-MSCs) (experimental group)

In this group, a combined interventions of both the PTE and WJ-MSCs will be administered.

### 2.4 Outcomes

On the day of the assessment, each participant performs the measures in the following order: cognitive function assessment, surveys/questionnaires, physical function assessment, biological assessment, and (MRI).

#### 2.4.1 Motor assessment measures

*2.4.1.1. Aerobic fitness* is assessed using the submaximal testing. In this test a seated recumbent stepper (Model SCIFIT-StepOne) will be used, which provides simultaneous movement of the upper and lower extremities in a continuous stepping motion. During the submaximal exercise test, the participants are asked to move their arms and legs in an alternating pattern at a constant speed of 100 steps per minute and resistance (Watts) will be increased every 3 minutes according to the total body recumbent stepper submaximal exercise test until 85% of age-predicted heart rate max is met or participant can no longer continue due to fatigue (test termination endpoint). During the test, heart rate is monitored using polar heart rate monitors. Ten seconds prior to the end of every stage, heart rate is taken and recorded. Once the test is finished, the participant will continue to step at a comfortable self-selected speed with minimal resistance for 2 minutes or until heart rate returns to near baseline levels.^[27]^ The outcome measure is predicted VO2 max calculated by a formula using the participant age, sex, weight, watts used, and HR.^[27]^

*2.4.1.2. Balance and mobility* are assessed using the following assessment tools:

1. 6-minute walk test will be used to assess physical endurance capacity.^[28]^ This test needs a hallway with 2 cones 15 meter apart to be performed. The participant walks between the 2 cones for 6 minutes. Distance is measured by number of laps to be completed.
2. The Arabic version of the dynamic gait index will be used to examine gait performance and the likelihood of falling ^[29]^. The dynamic gait index has been translated to several languages including the Arabic language.^[30]^
3. The Arabic version of the Berg balance scale will be used to assess static balance.^[31]^ It consists of 14 common functional activities that occur in everyday life.^[32]^
4. Timed-Up and go test is a test for assessing balance and walking ability.^[33]^ The participant is instructed to rise from a chair, walks 3 meters at a comfortable and safe pace, turns, walks back to the chair, and sits down. The time between the start of the test until the participant sits back on the chair is recorded and used as the outcome measure.
5. The Arabic version of the activities-specific balance confidence scale will be used to detect balance confidence.^[31]^ This scale was found to be both reliable and valid for the assessment of balance in PwMS.^[34,35]^
6. Barthel index is a self-reported questionnaire that evaluates the ability of individuals to perform activities of daily living and mobility activities ^[36]^. The BI is considered as a measure of disability that had good reliability and validity ^[36,37]^. The BI has been used with individuals with neurological disorders.^[38]^

2.4.1.3. *Upper extremity motor function (UEMF)* is assessed using the following tests:

1. Grip strength of the participants will be assessed using a hand held dynamometer (Model Jamar hydraulic hand dynamometer).^[39]^ Each participant will be instructed to squeeze the handle with maximum strength first on the dominant hand and then on the non-dominant hand. Three trials on each hand will be performed. The average maximum force exerted from the 3 trials will be used as a primary outcome measure.
2. 9-hole peg test is a specific test for finger dexterity.^[40]^ The participant is asked take pegs from a container, one by one and put them into holes. Then take the pegs back from the hole, one by one an out them into the container as quickly as possible. The time taken to finish the task will be recorded and used as an outcome measure. The test will be performed first on the dominant hand then on the non-dominant hand.

*2.4.1.4. Fall assessment*: the Arabic version of the falls efficacy scale-international will be used to assess fall.^[41]^ The FES-I is a self-report questionnaire that evaluates fear of falling.

#### 2.4.2 Non-Motor assessment measures:

*2.4.2.1. Depreesion* is assessed using the Arabic version of the Beck depression inventory-II.^[42]^ The Beck depression inventory-II measures how depression is manifested in behavior. It not only identifies varying levels of depression but is also able to indicate changes in intensity of depression over a period of time. Ratings are summed to provide a total score ranging from 0 to 63.^[43]^

*2.4.2.2. Fatigue* is assessed using the Arabic version of the modified fatigue impact scale (A-MFIS). The MFIS is a 21 item self-administered questionnaire that offers multidimensional assessment of the effects of fatigue in terms of physical, cognitive, and psychosocial functioning.^[44]^ The MFIS was translated into the Arabic language and was found valid and reliable tool to assess fatigue in people with MS.^[45]^

*2.4.2.3. Sleep quality* is assessed using the following tests:

1. The Arabic version of Pittsburgh sleep quality index will be used to assess sleep quality.^[46]^ Pittsburgh sleep quality index is a well-validated and reliable measure of global sleep quality which consists of 19 self-rated questions. These 19 questions form 7 component scores, each of which is rated on a scale of 0 to 3 with 0 indicating no sleep difficulty and 3 indicating severe sleep difficulties. The 7 component scores are then summed to form a single global score ranging from of 0 to 21. A global score of 5 or more reflects poor sleep quality for all age groups.^[47]^
2. The Arabic version of Epworth sleepiness scale (ESS) will be used to assess the daytime sleepiness.^[48]^ It consists of 8 items where the participant uses a 4-point Likert scale to rate how likely they would be to fall asleep in 8 different scenarios of daily activities.^[49]^

*2.4.2.4. Cognitive assessment* will be examined using the following measures:

1. The Stroop test is a tool performed as a measure of executive functioning that requires participants to inhibit the natural response (reading a word) and replace it with another response (saying a color) ^[50]^. A validated Arabic version will be used in this study.^[51]^
2. The symbol digit modalities test, oral form, is a measure of information processing speed that asks participants to quickly say the number that matches a corresponding symbol ^[52]^. The total correct responses in 90 seconds will be used as the outcome measure.^[52]^

*2.4.2.5. Pain* is assessed using the pain rating scale that was translated into the Arabic by the British pain society and recommended for use in populations where pain is a major issue.^[53]^

*2.4.2.6. Quality of life (QOL)* is assessed using the short form SF-36 (SF-36 version-1). The SF36 is a health-related quality of life measure.^[54]^ An Arabic version of the SF-36 has been validated.^[55]^

#### 2.4.3 Medical assessment

*2.4.3.1. Clinical disability assessment* is examined using the EDSS. The EDSS is the MS golden standard that assesses the degree of disability.^[56]^ The EDSS is performed by a trained internal medicine specialist under the supervision of the lead neurologist from Jordan University Hospital. It is a scale from 0 (very minimal signs of MS with no disability) to 10 (Death due to MS) with an increment of 0.5. An EDSS change of 0.5 or 1.0 point or more in either direction is considered as a significant change.

##### 2.4.3.1 Biological markers

Bio-specimens including blood and cerebrospinal fluid are collected from participants by a trained nurse. Inflammatory markers in the blood are examined using Multiplex ELISA technology. Neural regeneration markers will be measured in the CSF and blood collected. Tests will be performed by CTC biotechnologists. Moreover, the percentage of T, B and NK cells in the blood of participants will be calculated at the CTC flow cytometry lab by a dedicated biotechnologist. Additionally, the IgG levels in the CSF will be measured in a referral CAP accredited lab (MEDLAB).

The difference in concentrations compared to baseline measurements of biomarkers in the blood and CSF of patients in pg/mL enables the evaluation of the inflammatory and neural regeneration/degeneration status in response to interventions. While a change in the percentages of the different immune cells in participant's blood is an indication of immune-modulation outcome.

##### 2.4.3.2 Ophthalmological assessment

Visual evoked potential that measures the electrical conductivity is performed by a technologist at the CTC and supervised by the leading neurologist at the Jordan University Hospital.

Optical coherence tomography is conducted by technologist and supervised by an ophthalmologist at the Jordan University Hospital. A measurement of the retinal nerve fiber layer in um and comparison to the baseline measurements in both the poor eye and less affected eye would indicate the outcome of the Optical coherence tomography examination. While the increase or decrease in the speed of electrical conduction in milliseconds of the nerve fiber would be evaluated as the target heart rate range outcomes.

##### 2.4.3.3 Radiological Assessment

T2-weighted FLAIR and T1-weighted MPRAGE images are generated for all patients. The detailed imaging sequences protocol of the brain and those of the cervical and thoracic spine have been previously described.^[11]^ Fuzzy connections algorithm in JIM software is used to calculate lesion volume measurements.^[57]^

*2.4.3.5. MRI scanning* is performed at Farah hospital using a 3 Tesla (3T) imaging device to measure lesion load. Scheduling the MRI scanning will be on a separate occasion within 7 days from the day of the baseline assessment and prior to receiving the treatment.

### 2.5 Participant timeline

Eligible participants will be scheduled for their baseline assessments. They will undergo a comprehensive battery of measures including motor, non-motor, and medical assessments, which will take around 2 to 3 hours to complete. Part of the baseline assessment that include MRI imaging will be performed on another day within a week from the baseline assessment on a time convenient to the participant. Participants will repeat these measures at 3, 6, and 12 months post the baseline assessment (Fig. 1).

### 2.6 Allocation

Participants will be randomly allocated to 1 of 3 groups using a computer-generated software.

### 2.7 Blinding

This study is single-blinded where the researchers involved in the assessments are blinded to the allocation of the participants to each group.

### 2.8 Data management and confidentiality

All members of the research team work to protect the privacy of participants and the confidentiality of data. Personal identifiers are limited only on data collection hard copy forms and all data files are kept in a locked file cabinet. Each participant is given a unique identifier (symbol and number) and their electronic data is stored in university network drives and data collection occurs on an encrypted password-protected laptop computer. Data is available and accessed only by the study research personnel.

### 2.9 Statistical analysis

Statistical Package for the Social Sciences (SPSS) 23.0 (SPSS: Inc., Chicago, IL, USA) will be used to perform all statistical analysis and alpha is set at 0.05. Correlations will be utilized to assess the relationship between the outcome measures of interest. Changes in outcomes at the follow-up scores for within-group and between-group differences will be computed using analysis of variance. Effect sizes of all outcomes will be also computed based on the difference in the change score from base line to follow up.

### 2.10 Ethics and dissemination

This trial has been approved by the Ethics Review Committee at University of Jordan. Prior to participation in the study, written informed consent will be obtained from each participant. The research personnel involved in the study will carefully explain the consent form to each participant including potential risks and benefits of study participation. Any changes to the study protocol will require approval from University of Jordan Ethics Review Board.

The results of current trial will be published in peer-reviewed journals and presented in national and international scientific meetings and conferences to promote dissemination.

### 2.11 Declaration of interests

This trial is funded by the Deanship of Academic Research at University of Jordan. The authors declare that they have no competing interests.

## 3 Discussion

In this study we will investigate the combined effect of stem cell therapy and physical therapy exercises in a wide range of MS symptoms. Moreover, we will compare the effect of a combined therapy to the separate effect of each therapeutic intervention alone. This will help health care professionals dealing with PwMS to have a better understanding of the combined versus separate effects of each therapeutic intervention. The outcomes from this study may help in reducing the cost associated with the life-time care of these individuals worldwide. More importantly, this study may help improving patients’ QOL and reducing their disability. For rehabilitation specialists, neurologists, and regenerative medicine specialists, the outcomes of this trial would clarify the added value of the regenerative interventions with the physical therapy exercise on different symptoms that PwMS suffer from. This will allow them to have a better understanding of this combination to answer PwMS questions that they usually have. Moreover, the combination between biological interventions and having the patients physically active through regular and progressive exercises would emphasize the benefits that the patients may get from both techniques. The expected benefits from both interventions could lead to a medical breakthrough in the treatment of PwMS. This would have positive socio-economic impact, including reducing the treatment cost related to the chronic nature of the disease and getting this active segment of the society back to the work market.

## Acknowledgments

Authors would like to thank the CTC personnel, research assistants, and patients for their commitment to this trial.

## Author contributions

Alia Alghwiri and Fatima Jamali have equal contribution and both of them deserve to be first author.

**Conceptualization:** Alia Alghwiri, Fatima Jamali.

**Data curation:** Alia Alghwiri, Fatima Jamali, Mayis Aldughmi.

**Funding acquisition:** Alia Alghwiri, Fatima Jamali, Abdalla Awidi.

**Investigation:** Alia Alghwiri, Fatima Jamali, Mayis Aldughmi, Dana Alhattab, Ali Al-Radaideh, Abdalla Awidi.

**Methodology:** Alia Alghwiri, Fatima Jamali, Mayis Aldughmi, Hanan Khalil, Alham Al-Sharman, Dana Alhattab, Ali Al-Radaideh, Abdalla Awidi.

**Project administration:** Fatima Jamali, Abdalla Awidi.

**Supervision:** Abdalla Awidi.

**Writing – original draft:** Alia Alghwiri, Fatima Jamali, Mayis Aldughmi, Hanan Khalil, Alham Al-Sharman.

**Writing – review & editing:** Alia Alghwiri, Fatima Jamali, Abdalla Awidi.

## Supplementary Material

Supplemental Digital Content

## Supplementary Material

Supplemental Digital Content

## Supplementary Material

Supplemental Digital Content

## Supplementary Material

Supplemental Digital Content
