# Supplementary material for: The effect of stem cell therapy and comprehensive physical therapy in motor and non-motor symptoms in patients with multiple sclerosis: A comparative study
Source: Medicine (Baltimore). 2020 Aug 21;99(34):e21646. doi: 10.1097/MD.0000000000021646 (PMC7447403; doi:10.1097/MD.0000000000021646)
Supplement: Supplemental Digital Content [file medi-99-e21646-s001.docx]

**The effect of stem cell therapy and comprehensive physical therapy in motor and non-motor symptoms in patients with multiple sclerosis: A comparative study**

Alia A. Alghwiri, PhD^a^, Fatima Jamali, PhD^b^, Mayis Aldughmi, PhD^a^, Hanan Khalil, PhD^c^, Alham Al-Sharman, PhD^c^, Dana Alhattab, PhD^b^, Ali Al-Radaideh, PhD^d^, Abdalla Awidi, PhD ^b,e*^

**1. Supplemental Digital Content (Appendix 1):** Resistance and flexibility exercise program

1. **Stage I (First 2 months total 16 sessions)**
   - The frequency is 1 set of 10 Reps
   - The resistance is either 0.5 kg cuff weight or light Thera band
   - Resting period is 2 minutes between each exercise
   - **Exercises: (Participant/Examiner choose five exercises every session, must perform the remaining five the next session)**
2. Bridges with 2 legs (Each Rep for 20 sec)
3. Table tops (1 leg out/ 0.5 kg ankle weight or light Thera band/ each leg 1 set/ each Rep 5 sec)
4. Planks on elbows (1 arm stretch out/ 0.5 kg wrist weight/ each arm 1 set/ each Rep hold 5 sec)
5. Squats with 0.5 kg wrist weights (arms outstretched in front of body)
6. Lunges with light Thera band (Each leg 1 set)
7. Reverse lunges with light Thera band (Each leg 1 set)
8. Hip abduction standing at a desk/chair: lift one leg out to the side with light Thera band (Each leg 1 set)
9. Warrior I pose (Alternate sides each 1 set)
10. Step ups/step downs using step stool lowest height/ 0.5 kg ankle weight/ each leg 1 set
11. Skip jumps feet together
12. **Stage II (Months 3-4 total 16 sessions)**
    - The frequency is 1 set of 15 Reps
    - The resistance is either 1 kg cuff weight or medium Thera band
    - Resting period is 1 minute between each exercise
    - **Exercises: (Participant/Examiner choose five exercises every session, must perform the remaining five the next session)**
13. Bridges with 2 legs holding ball with two hands (Each Rep for 20 sec)
14. Table tops (1 leg out with 1 kg ankle weight or medium Thera band/ each leg 1 set/ each Rep 5 sec)
15. Planks on elbows (1 arm stretch out/ 1 kg wrist weight/ each arm 1 set/ each Rep hold 5 sec)
16. Squats with 1 kg wrist weights (arms outstretched in front of body)
17. Lunges with medium Thera band (Each leg 1 set)
18. Reverse lunges with medium Thera band (Each leg 1 set)
19. Hip abduction standing at a counter: lift one leg out to the side a desk/chair: lift one leg out to the side with medium Thera band (Each leg 1 set)
20. Warrior II pose (Alternate sides each 1 set)
21. Step ups/step downs using step stool lowest height/ 1 kg ankle weight/ each leg 1 set
22. Skip jumps alternating feet/ knees high
23. **Stage III (Months 5-6 total 16 sessions)**
    - The frequency is 2 sets of 15 Reps
    - The resistance is either 1.5 kg cuff weight or heavy Thera band
    - Resting period is 1 minute between each exercise
    - Resting period is 2 minutes between each set
    - **Exercises: (Participant/Examiner choose five exercises every session, must perform the remaining five the next session)**
    1. Bridges with 1 leg (Each leg 2 sets/ Each Rep for 20 sec)
    2. Table tops (1 leg out with 1.5 kg ankle weight or heavy Thera band/ each leg 1 set/ each Rep 5 sec)
    3. Planks on elbows (1 arm stretch out/ 1.5 kg wrist weight/ each arm 2 sets/ each Rep hold 5 sec)
    4. Squats with 1.5 kg wrist weights (arms outstretched in front of body)
    5. Lunges with heavy Thera band (Each leg 2 sets)
    6. Reverse lunges with heavy Thera band (Each leg 2 sets)
    7. Hip abduction standing at a counter: lift one leg out to the side a desk/chair: lift one leg out to the side with heavy Thera band (Each leg 1 set)
    8. Warrior III pose (Alternate sides each 2 set)
    9. Step ups/step downs using step stool highest height/ 1.5 kg ankle weight/ each leg 2 sets
    10. Skip jumps feet together above an obstacle
