# Supplementary material for: The effect of stem cell therapy and comprehensive physical therapy in motor and non-motor symptoms in patients with multiple sclerosis: A comparative study
Source: Medicine (Baltimore). 2020 Aug 21;99(34):e21646. doi: 10.1097/MD.0000000000021646 (PMC7447403; doi:10.1097/MD.0000000000021646)
Supplement: Supplemental Digital Content [file medi-99-e21646-s002.docx]

**The effect of stem cell therapy and comprehensive physical therapy in motor and non-motor symptoms in patients with multiple sclerosis: A comparative study**

Alia A. Alghwiri, PhD^a^, Fatima Jamali, PhD^b^, Mayis Aldughmi, PhD^a^, Hanan Khalil, PhD^c^, Alham Al-Sharman, PhD^c^, Dana Alhattab, PhD^b^, Ali Al-Radaideh, PhD^d^, Abdalla Awidi, PhD ^b,e*^

**2. Supplemental Digital Content (Appendix 2):** Balance exercise program

1. **Stage I (1^st^ 2 months total 16 sessions)**
   - The frequency as indicated in each exercise
   - The intensity as indicated in each exercise
   - Resting period is 1 minute between each exercise
   - **Exercises: (Participant/Examiner choose five exercises every session, must perform the remaining five the next session)**
2. Stand on foam: feet shoulder width apart/ eyes open for 20 sec/ 5 Reps
3. Stand on foam: feet in tandem, right foot leading/ eyes open for 20 sec/ 5 Reps
4. Stand on foam: feet in tandem, left foot leading/ eyes open for 20 sec/ 5 Reps
5. Walk on a line on floor (2 meters)/ heel to toe/ alternating legs/ 10 Reps
6. Walk (2 meters) turning head sideways and up and down/ eyes open/10 Reps
7. Walk backwards (2 meters)/ 10 Reps
8. Walk over obstacles (5- 10 cm, 2 meters)/ 10 Reps
9. Walk on uneven surface (put sandbags or cuff weight under matt)/ eyes open/ 10 Reps
10. Four-square stepping/ both directions/ 10 Reps
11. Grapevine stepping (crossing legs- 2 meters)/ 10 Reps
12. Sit to stand from chair with arms outstretched/ 10 Reps
13. Sit to stand from Swiss ball/ eyes open/ 10 Reps
14. Play catch with small ball from standing feet shoulder width apart/ 10 Reps
15. Stand on 1 leg/ eyes open for 20 sec/ 5 Reps/ alternate legs
16. Stand on balance board eyes open for 20 sec/ 5 Reps
17. **Stage II (Months 3-4 total 16 sessions)**
    - The frequency as indicated in each exercise
    - The intensity as indicated in each exercise
    - Resting period is 1 minute between each exercise
    - **Exercises: (Participant/Examiner choose five exercises every session, must perform the remaining five the next session)**
18. Stand on foam: feet shoulder width apart/ eyes closed for 20 sec/ 5 Reps
19. Stand on foam: feet in tandem, right foot leading/ eyes closed for 20 sec/ 5 Reps
20. Stand on foam: feet in tandem, left foot leading/ eyes closed for 20 sec/ 5 Reps
21. Walk on a line on floor (2 meters) counting backwards: days of the week, numbers in 10s, or months of the year/ heel to toe/ alternating legs/ 10 Reps
22. Walk (2 meters) turning head sideways and up and down/ eyes closed/ 10 Reps
23. Walk backwards (2 meters)/ counting backwards: days of the week, numbers in 10s, or months of the year/10 Reps
24. Walk over obstacles (5- 10 cm, 2 meters)/ counting backwards: days of the week, numbers in 10s, or months of the year/ 10 Reps
25. Walk on uneven surface (put sandbags or cuff weight under matt)/ eyes closed/ 10 Reps
26. Four-square stepping/ both directions/ counting backwards: days of the week, numbers in 10s, or months of the year/ 10 Reps
27. Grapevine stepping (crossing legs- 2 meters)/ counting backwards: days of the week, numbers in 10s, or months of the year/ 10 Reps
28. Sit to stand from chair with arms outstretched on foam/ narrow base of support/ 10 Reps
29. Sit to stand from Swiss ball/ eyes closed/ 10 Reps
30. Play catch with small ball from narrow base of support/ 10 Reps
31. Stand on 1 leg on foam/ eyes open for 20 sec/ 5 Reps/ alternate legs
32. Stand on balance board/ eyes closed for 20 sec/ 5 Reps
33. **Stage III (Months 5-6 total 16 sessions)**
    - The frequency as indicated in each exercise
    - The intensity as indicated in each exercise
    - Resting period is 1 minute between each exercise
    - **Exercises: (Participant/ Examiner choose five exercises every session, must perform the remaining five the next session)**
34. Stand on foam: feet shoulder width apart/ eyes closed/ holding tray with glasses for 20 sec/ 5 Reps
35. Stand on foam: feet in tandem, right foot leading/ eyes closed/ holding tray with glasses for 20 sec/ 5 Reps
36. Stand on foam: feet in tandem, left foot leading/ eyes closed/ holding tray with glasses for 20 sec/ 5 Reps
37. Walk on a line on floor (2 meters) counting backwards: days of the week, numbers in 10s, or months of the year/ holding tray with glasses/ heel to toe/ alternating legs/ 10 Reps
38. Walk (2 meters) turning head sideways and up and down/ eyes closed/ holding shopping bags/10 Reps
39. Walk backwards (2 meters)/ counting backwards: days of the week, numbers in 10s, or months of the year/ holding shopping bags/ 10 Reps
