# Supplementary material for: The effect of stem cell therapy and comprehensive physical therapy in motor and non-motor symptoms in patients with multiple sclerosis: A comparative study
Source: Medicine (Baltimore). 2020 Aug 21;99(34):e21646. doi: 10.1097/MD.0000000000021646 (PMC7447403; doi:10.1097/MD.0000000000021646)
Supplement: Supplemental Digital Content [file medi-99-e21646-s003.docx]

**The effect of stem cell therapy and comprehensive physical therapy in motor and non-motor symptoms in patients with multiple sclerosis: A comparative study**

Alia A. Alghwiri, PhD^a^, Fatima Jamali, PhD^b^, Mayis Aldughmi, PhD^a^, Hanan Khalil, PhD^c^, Alham Al-Sharman, PhD^c^, Dana Alhattab, PhD^b^, Ali Al-Radaideh, PhD^d^, Abdalla Awidi, PhD ^b,e*^

**3. Supplemental Digital Content (Appendix 3):** The protocol for the home exercise program (HEP) instructional sessions.

1. **Working through the DVD**
2. Revising the different sections of the exercise DVD
3. Explaining the use of the counter and the background music
4. Revising the correct posture
5. Revising the list of the precautions
6. While watching the DVD ask the following questions:
   - Where in home can you see yourself doing this?
   - Which chair would you use?
   - How might this fit into your everyday routine?
7. Making sure that the participant at home has got enough space and safe environment to perform the exercises
8. Picking up with the participants the exercises to start with (The subject can choose to do all exercises; subject must NOT performs less than half of the exercises in the manual)
9. Ensuring the correct execution of exercises; Performing the chosen exercises with the participant
10. Exercises should be performed two times/week
11. Participants are encouraged to exercise every other day to allow for a day rest in between.
12. Examiner explain to the participant how to document their compliance to the program using the weekly log
13. Examiner hand in the DVD, manual, and weekly exercise log to the participant

Each participant is followed up on a monthly basis using a phone call to assess compliance and document barriers to exercise.
