# Supplementary material for: The effect of stem cell therapy and comprehensive physical therapy in motor and non-motor symptoms in patients with multiple sclerosis: A comparative study
Source: Medicine (Baltimore). 2020 Aug 21;99(34):e21646. doi: 10.1097/MD.0000000000021646 (PMC7447403; doi:10.1097/MD.0000000000021646)
Supplement: Supplemental Digital Content [file medi-99-e21646-s004.docx]

**The effect of stem cell therapy and comprehensive physical therapy in motor and non-motor symptoms in patients with multiple sclerosis: A comparative study**

Alia A. Alghwiri, PhD^a^, Fatima Jamali, PhD^b^, Mayis Aldughmi, PhD^a^, Hanan Khalil, PhD^c^, Alham Al-Sharman, PhD^c^, Dana Alhattab, PhD^b^, Ali Al-Radaideh, PhD^d^, Abdalla Awidi, PhD ^b,e*^

| **Exercise Diary (week 1) Week start date: Week end date:**  **1- Please check when exercise is completed (record the number of repetition for each exercise)** | | | | | | | | | | | | |
| --- | --- | --- | --- | --- | --- | --- | --- | --- | --- | --- | --- | --- |
|  |  |  |  |  |  |  |  |  |  |  |  |  |
| **1** |  |  |  |  |  |  |  |  |  |  |  |  |
| **2** |  |  |  |  |  |  |  |  |  |  |  |  |
|  |  |  |  |  |  |  |  |  |  |  |  |  |
| **1** |  |  |  |  |  |  |  |  |  |  |  |  |
| **2** |  |  |  |  |  |  |  |  |  |  |  |  |

**4. Supplemental Digital Content (Appendix 4):** The exercise diary for the home exercise program (HEP).
